# Dopamine dynamics in nucleus accumbens across reward-based learning of goal-directed whisker-to-lick sensorimotor transformations in mice

**DOI:** 10.1016/j.heliyon.2024.e37831

**Published:** 2024-09-11

**Authors:** Jun Huang, Sylvain Crochet, Carmen Sandi, Carl C.H. Petersen

**Affiliations:** a Laboratory of Sensory Processing, Brain Mind Institute, Faculty of Life Sciences, Ecole Polytechnique Fédérale de Lausanne (EPFL), Lausanne, Switzerland; b Laboratory of Behavioral Genetics, Brain Mind Institute, Faculty of Life Sciences, Ecole Polytechnique Fédérale de Lausanne (EPFL), Lausanne, Switzerland

## Abstract

The synaptic and neuronal circuit mechanisms underlying reward-based learning remain to be fully determined. In the mammalian brain, dopamine release in nucleus accumbens is thought to contribute importantly to reward signals for learning and promoting synaptic plasticity. Here, through longitudinal fiber photometry of a genetically-encoded fluorescent sensor, we investigated dopamine signals in the nucleus accumbens of thirsty head-restrained mice as they learned to lick a liquid reward spout in response to single deflections of the C2 whisker across varying reward contingencies. Reward delivery triggered by well-timed licking drove fast transient dopamine increases in nucleus accumbens. On the other hand, unrewarded licking was overall associated with reduced dopamine sensor fluorescence, especially in trials where reward was unexpectedly omitted. The dopamine reward signal upon liquid delivery decreased within individual sessions as mice became sated. As mice learned to lick the reward spout in response to whisker deflection, a fast transient sensory-evoked dopamine signal developed, correlating with the learning of the whisker detection task across consecutive training days, as well as within single learning sessions. The well-defined behavioral paradigm involving a unitary stimulus of a single whisker as a reward-predicting cue along with precisely quantified licking allows untangling of sensory, motor and reward-related dopamine signals and how they evolve across the learning of whisker-dependent goal-directed sensorimotor transformations. Our longitudinal measurements of dopamine dynamics across reward-based learning are overall consistent with the hypothesis that dopamine could play an important role as a reward signal for reinforcement learning.

## 1 Introduction

Reward-based learning drives important aspects of animal behavior, but the precise changes and the underlying mechanisms in the mammalian brain remain to be fully understood for even the simplest goal-directed sensorimotor transformations. Investigations in mice have begun to shed light on the neuronal circuits contributing to various sensory-guided decision-making tasks [[1], [2], [3]]. Studying head-restrained mice offers precise stimulus control together with the possibility of making detailed measurements and manipulations of neuronal activity, helping advance causal understanding. Mice obtain important sensory information about their immediate surroundings from their array of facial whiskers and head-restrained mice can learn various whisker-dependent tasks including the detection of brief single whisker deflections [[4], [5], [6]], discrimination of different textures [[7], [8], [9]], object localization [10,11] and shape discrimination [12,13]. In these tasks, mice are typically water-restricted and learn through trial-and-error feedback to obtain water by licking a reward spout in response to the appropriate whisker sensory input. Many regions of the mouse brain are thought to contribute to such goal-directed whisker-to-lick sensory-to-motor transformations, including various cortical regions [[14], [15], [16], [17], [18], [19], [20], [21]], higher-order thalamus [[22], [23], [24]], and the striatum [[25], [26], [27]], but the precise changes in neuronal circuitry driving the reward-based learning remain to be fully determined.

The most prominently described reward signal in the mammalian brain is a transient increase in the firing of midbrain dopamine neurons in response to unexpected rewards or reward-predicting cues [28,29]. The midbrain dopamine neurons project strongly to the striatum, with dopamine neurons in the substantia nigra pars compacta mainly innervating the dorsal striatum and dopamine neurons in the ventral tegmental area (VTA) mainly innervating the ventral striatum, largely involving the nucleus accumbens. The dopamine reward signal has been proposed to contribute to learning by acting on dopamine receptors expressed on striatal neurons to gate the synaptic plasticity of glutamatergic inputs. In particular, dopamine acting on dopamine type 1 receptors (D1Rs) expressed on medium spiny projection neurons (MSNs) has been shown to enhance the induction of long-term potentiation of glutamatergic inputs to these neurons [[30], [31], [32]]. The dopamine reward signal could therefore act to enhance the strength of excitatory input arriving on D1R-expressing MSNs (D1R-MSNs) around the time of reward delivery. This could lead to behavioral reinforcement because the activity of D1R-MSNs has been found to enhance action initiation [[33], [34], [35], [36]]. Through modulating synaptic plasticity, the dopamine reward signal might thus help link reward-predicting stimuli and action initiation to obtain more rewards. Support for this hypothesis in the context of whisker-dependent task-learning, has come from in vivo membrane potential recordings from identified MSNs in dorsolateral striatum comparing naïve mice to expert mice that have learned to lick a reward spout in response to whisker deflection [25,26,37]. In these experiments, an early whisker sensory response was specifically enhanced across learning in D1R-MSNs, but not in D2R-MSNs [25]. Furthermore, optogenetic stimulation of D1R-MSNs but not D2R-MSNs drove a licking response in expert mice [26]. Learning to lick in response to a whisker deflection might therefore involve dopamine-dependent strengthening of whisker-sensory input to D1R-MSNs. The enhanced output of the D1R-MSNs in dorsolateral striatum might inhibit the tonically active neurons of the substantia nigra pars reticulata causing disinhibition of downstream targets such as thalamus and brainstem motor areas thus enhancing the initiation of licking. Although these data are consistent with the hypothesis that dopamine reward signals might drive striatal plasticity for reinforcement learning, to date dopamine signals have not yet been investigated in the learning of whisker-dependent tasks. Recent advances in the development of genetically-encoded fluorescent sensors, such as dLight [38] and GRAB-DA [39], allow the measurement of changes in dopamine concentration with high temporal resolution through fiber photometry. Here, in this study, we therefore optically measured dopamine dynamics longitudinally as thirsty mice learned to lick a water reward spout in response to whisker deflection, finding a prominent phasic dopamine increase in response to reward delivery, and also in response to the reward-predicting whisker deflection as mice learned the task across days and within single sessions.

## 2 Results

### 2.1 Dopamine measurements across reward-based whisker-to-lick learning

**Fig. 1:**
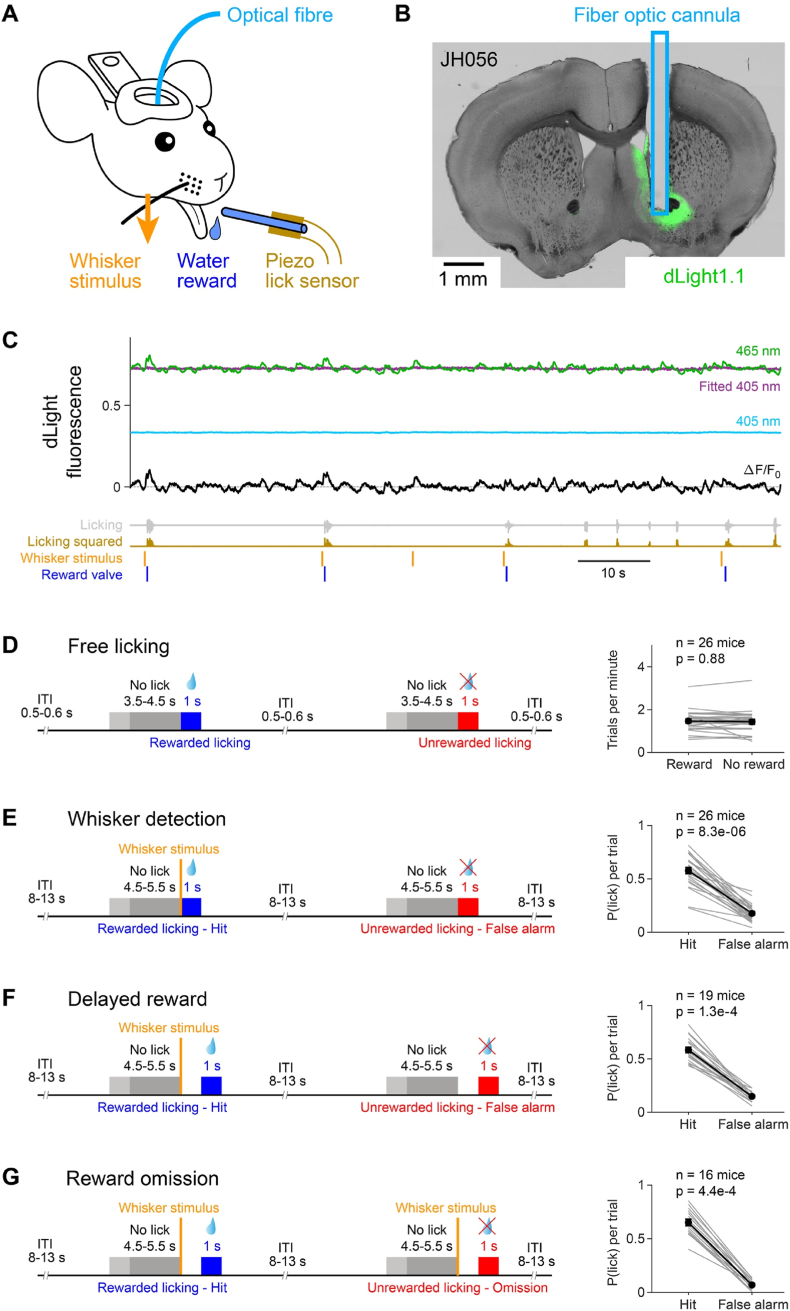
Measurement of dopamine dynamics during behavior. (**A**) Adenoassociated virus was injected into the nucleus accumbens to express the genetically-encoded fluorescent dopamine sensor dLight1.1 and fluorescence was measured during head-restrained behavior through an implanted fiber optic cannula coupled to a fiber photometry system. A reward spout was placed in front of the mouse and tongue-spout contacts were measured using piezofilm attached to the reward spout. Liquid rewards of ∼5 μl were delivered by opening a valve at appropriate moments controlled by a computer depending upon the deflection of the C2 whisker, the timing of licking, and the current task reward contingencies. (**B**) A coronal section of an example mouse JH056 near the virus injection site. The greyscale image shows the bright field contrast of the 100 μm-thick section, and the green color indicates the GFP fluorescence from the dLight sensor. The location of the fiber optic cannula is schematically indicated in cyan, with the fiber tip near the nucleus accumbens. (**C**) Example dLight fluorescence and licking measurements from mouse JH056 during the whisker detection task. Fluorescence was excited by 465 nm (green, dopamine sensitive wavelength) and 405 nm (cyan, dopamine insensitive wavelength). The 405 nm-excited fluorescence was scaled to the 465 nm-excited fluorescence (purple, fitted 405 nm). dF/F_0_ was calculated as the ratio of 465 nm-excited fluorescence to the fitted 405 nm-excited fluorescence minus 1 (black). The raw licking piezo signal (grey) and the licking power computed as the square of the licking signal (brown) are shown below together with the timing of whisker stimuli (orange vertical bars) and opening of the reward valve (blue vertical bars). (**D**) In the Free licking task mice, thirsty mice were rewarded with 50 % probability if they initiated licking within a 1-s reward window, which was preceded by a 3.5–4.5 s period during which the mice were not allowed to lick. Most mice learned to lick for reward and retrieved on average 1–2 rewards per minute (*Right*, n = 26 mice, Wilcoxon signed-rank test). (**E**) In the Whisker detection task, the same mice as above were rewarded for licking in the 1-s period immediately after a single brief deflection of the C2 whisker. The whisker stimulus was only presented in half of the trials, allowing us to monitor spontaneous lick initiation rates in False alarm trials. Mice were required not to lick in the 4.5–5.5 s period preceding the whisker stimulus. Over the first 3 days of training in the whisker detection task, mice learned to lick significantly more on trials with a whisker stimulus (Hit rate) compared to trials without a whisker stimulus (False alarm rate) (*Right*, n = 26 mice, Wilcoxon signed-rank test). (**F**) A subset of the mice was further trained in the Delayed reward task. Reward contingency was the same as for the Whisker detection task, except the reporting window was delayed by 1 s. Mice were free to lick during the delay period, but only licking during the delayed reporting window starting a second after whisker stimulation was rewarded. Over the first 3 days of training in the Delayed reward task, mice licked significantly more on trials with a whisker stimulus (Hit rate) compared to trials without a whisker stimulus (False alarm rate) (*Right*, n = 19 mice, Wilcoxon signed-rank test). (**G**) A further subset of mice participated in the Reward omission task, which was identical to the Delayed reward task, except that reward was omitted in approximately half of the Hit trials. A single session of the Reward omission task was carried out and mice licked significantly more on trials with a whisker stimulus (Hit rate) compared to trials without a whisker stimulus (False alarm rate) (*Right*, n = 16 mice, Wilcoxon signed-rank test).

In order to optically measure dopamine signals, we injected adenoassociated virus (AAV) encoding the genetically-encoded fluorescent dopamine sensor dLight1.1 [38] into the nucleus accumbens of the left brain hemisphere of mice. A fiber optic cannula was subsequently implanted with its tip ∼100 μm above the injection site, allowing chronic dopamine measurements across days by plugging it into a fiber optic patch cable connected to a fiber photometry system at the beginning of each behavioral session (Fig. 1A) [40]. At the end of the behavioral experiments, the fixed brains were cut into 100-μm thick coronal sections and the location of the end of the fiber optic cannula was identified as well as the location of the dLight fluorescence (Fig. 1B). Altogether in this study, we analyzed 26 mice for which the optic fiber cannula was correctly implanted to measure dLight signals in nucleus accumbens with high signal-to-noise ratio.

Fluorescence was excited by 405 nm and 465 nm sinusoidally modulated light at ∼200 Hz and ∼500 Hz respectively, and bandpass-filtered 500–550 nm emitted light was collected and demodulated [41]. The dopamine-insensitive dLight fluorescence excited at 405 nm was linearly scaled to the dopamine-sensitive dLight fluorescence excited at 465 nm. The scaled 405 nm excited signal was subtracted from the 465 nm excited signal and the ratio, dF/F_0_, was calculated (Fig. 1C). In order to compare task-related signals across mice and allow longitudinal comparisons across the learning of various whisker-to-lick tasks described below, the dF/F_0_ signal was further divided by the standard deviation of the trial-averaged signals across the different trial types and tasks to compute a z-scored dF/F_0_ signal with a single normalization factor for each mouse applied to all recording days.

Head-restrained behavioral experiments began after allowing at least 4 weeks for the dLight to express. In the first experiment, water-restricted mice were presented with a reward spout that would deliver a ∼5 μl drop, triggered by licking detected by piezo film attached to the spout. The piezo film provides an online voltage reading largely-related to the forces on the spout upon tongue contact. No additional sensory stimuli were delivered in this ‘Free licking’ task (Fig. 1D). Although not explicitly indicated to the mouse, the task had a trial structure. Trials were separated by a 0.5–0.6 s inter-trial interval, and if the mouse licked in the subsequent 3.5–4.5 s ‘No lick’ period preceding the 1-s reporting period, then the trial was aborted. Reward was only delivered upon licking during the 1-s reporting period in half of these trials (i.e. with a 50 % probability) to give two trial types of interest: rewarded and unrewarded licking. dLight measurements from 26 mice were analyzed during the second day of Free licking, when the mice were skilled in licking the spout and highly motivated by thirst. On average, mice successfully initiated licking in the reporting period at a rate of 1.47 ± 0.10 rewarded trials per minute and 1.44 ± 0.11 unrewarded trials per minute (mean ± sem, n = 26 mice).

After 2 days of the Free licking task, the mice began to be trained in the ‘Whisker detection’ task (Fig. 1E). In this task, the C2 whisker on the right side was briefly deflected to indicate the onset of a 1-s reward window during which licking was rewarded. In half of the trials, the whisker stimulus was not present and licking was not rewarded. Trials with and without whisker stimulus were randomly interleaved and were separated by an 8–13 s intertrial interval and a 4.5–5.5 s No-lick period during which mice were required not to lick in order to initiate a new trial. This gives rise to four trial types of interest: i) Hit trials with a whisker stimulus, licking and reward; ii) Miss trials with a whisker stimulus but no licking and therefore no reward; iii) False alarm trials without whisker stimuli but with spontaneous licking which was not rewarded; and iv) Correct rejection trials with no whisker stimulus, no licking and no reward. The same mice as before were trained over at least 3 days in the Whisker detection task and dLight photometry was carried out for each session. Most mice learned the task within 3 days, but some mice were trained for further days to reach expert levels before moving to the next task. Averaged across the first 3 days of training in the Whisker detection task, mice correctly licked to obtain reward in 57.8 ± 2.9 % of trials with a whisker deflection (Hit rate) and incorrectly licked without obtaining reward in 17.7 ± 1.6 % of trials without a whisker deflection (False alarm rate), which were significantly different (p = 9.3 x 10^−6^, Wilcoxon signed-rank test, n = 26 mice).

A subset of the 26 dLight-expressing mice trained in the Whisker detection task was further trained in the ‘Delayed reward’ task, which was the same as the Whisker detection task in every respect except that the reward window was delayed by 1 s (Fig. 1F). The mice were allowed to lick in the delay period between the whisker stimulus and the opening of the reward window, but the trigger for opening the reward valve required licking in the reward window. The same four trial types were analyzed as above: Hit, Miss, False alarm and Correct rejection. Mice were trained for three days in the Delayed reward task. Averaged across the three days of the Delayed reward task, the Hit rate was 58.3 ± 2.6 % and the False alarm rate was 14.9 ± 1.1 %, which were significantly different (p 1.3 x 10^−4^, Wilcoxon signed-rank test, n = 19 mice).

In the final experiment, a subset of the 19 mice trained in the Delayed reward task was subjected to a ‘Reward omission’ task (Fig. 1G). The task design was identical to the Delayed reward task, except reward was withheld in approximately half of the trials in which a whisker stimulus was delivered despite licking in the reporting period (ratio of rewarded Hit to unrewarded Hit trials was 1.19 ± 0.10, n = 16 mice). This gives rise to five trial types of interest: i) Rewarded Hit trials including a whisker stimulus, licking during the reporting window and reward delivery; ii) Unrewarded Hit trials with a whisker stimulus, licking during the reporting window but no reward delivery; iii) Miss trials with a whisker stimulus, but no licking during the reporting window and no reward delivery; iv) False alarm trials with no whisker stimulus, but licking in the reporting window and no reward; and v) Correct rejection trials with no whisker stimulus, no licking and no reward. In this task, a single session of dLight recordings were made. For the Reward omission task, the Hit rate (including both rewarded and unrewarded Hit trials) was 65.2 ± 3.0 % and the False alarm rate (licking during the reporting period in the absence of a whisker stimulus) was 7.0 ± 1.2 %, which were significantly different (p = 4.4 x 10^−4^, Wilcoxon signed-rank test, n = 16 mice).

We also tested the causal role of the nucleus accumbens in the execution of the Whisker detection task in a separate group of 6 mice, trained similarly to perform the task, but not injected with AAV to express dLight. We successively injected muscimol to inactivate, and Ringer's solution as a control, into the nucleus accumbens bilaterally on alternating days. Muscimol injection – but not injection of Ringer's solution – caused a pronounced and significant decrease in performance (Fig. S1), suggesting that neuronal activity in nucleus accumbens contributes to execution of the Whisker detection task.

### 2.2 Task-related dLight signals

**Fig. 2:**
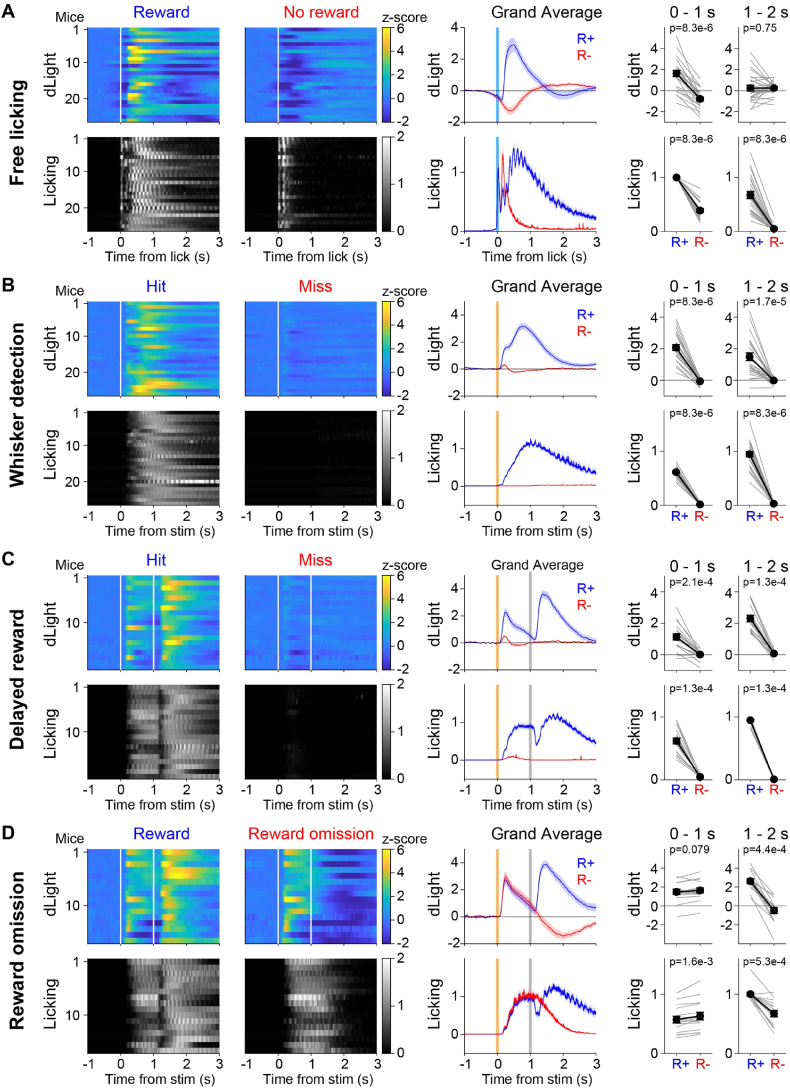
Task-related licking and dLight signals in nucleus accumbens. (**A**) Color-coded trial-averaged dLight fluorescence dynamics aligned to licking onset (white vertical bar at time 0 s) for each of the 26 mice recorded in the Free licking task for rewarded and unrewarded licking (upper left). For the same mice the licking power (normalized squared voltage measured by the piezo film) is indicated in grey for rewarded and unrewarded licking (lower left). The grand average dLight and licking dynamics across mice together with sem shading for rewarded (R+, blue) and unrewarded (R-, red) licking (center right). The average dLight fluorescence and licking in the 0–1 s and 1–2 s periods following lick onset was quantified for each mouse (grey lines) together with the mean ± sem (black lines and filled circles with error bars; Wilcoxon signed-rank test). (**B**) Similar to panel A, but for the Whisker detection task averaged across the three days of training and aligned to the time of the whisker deflection at time 0 s and contrasting Hit trials in which the mice received whisker stimulus, licked the spout and received reward with Miss trials in which the same whisker stimulus was delivered but the mouse failed to lick and therefore received no reward. (**C**) Same as panel B, except now showing the subset of 19 mice that carried out the Delayed reward task. As above, the Hit trials and Miss trials were aligned to whisker stimulus delivered at time 0 s. The second vertical white line at time 1 s indicates the beginning of the reporting period, when mice were rewarded for licking in Hit trials. (**D**) Same as panel C, but now for the 16 mice that underwent the Reward omission task, comparing Rewarded Hit trials and Reward omission trials which were unrewarded Hit trials.

We found prominent task-related dLight signals in each of the four behavioral paradigms investigated (Fig. 2). In the Free licking task, we observed a clear difference between rewarded and unrewarded licking in the reporting period when aligning dLight signals to the onset of licking (Fig. 2A). On average, licking onset was preceded by a small initial decrease in dLight fluorescence over ∼500 ms before the tongue contacted the reward spout at time 0 ms. If the licking was unrewarded, the dLight fluorescence continued to decrease until reaching a trough ∼400 ms later and then gradually returning to baseline. However, rewarded licking was followed after a delay of ∼100 ms by a fast-rising increase in dLight fluorescence peaking at ∼500 ms after tongue-spout contact and decaying over approximately 1 s. Quantified as the average z-score dF/F_0_ over the 1-s period after tongue-spout contact relative to a baseline 1–0.5 s before tongue-spout contact, rewarded licking evoked a significantly higher dLight signal compared to unrewarded licking (Rewarded 1.63 ± 0.29, Unrewarded −0.79 ± 0.17; p = 8.3 x 10^−6^, Wilcoxon signed-rank test; n = 26 mice). Computed across the next 1-s period, the dLight signal had largely returned to baseline values without a difference between rewarded and unrewarded trials (Rewarded 0.24 ± 0.27, Unrewarded 0.25 ± 0.13; p = 0.75, Wilcoxon signed-rank test; n = 26 mice). Mice also licked for a much longer duration in rewarded compared to unrewarded trials.

In the Whisker detection task, there were also clear differences comparing rewarded Hit trials with the other trial types, including Miss trials, in which the whisker deflection was delivered but the mice failed to lick in the appropriate reward window and thus no water was given (Fig. 2B). Averaged across the first three days of training in the Whisker detection task, whisker deflection evoked a large, apparently biphasic increase in dLight fluorescence in Hit trials, whereas only a small, brief transient increase in dLight fluorescence followed by a small reduction below baseline was found in Miss trials. Quantified across the 1 s period following whisker deflection relative to a baseline 0.5–0 s before whisker stimulus, there was a significant difference comparing dLight signals in Hit and Miss trials (Hit 2.07 ± 0.17, Miss −0.07 ± 0.04; p = 8.3 x 10^−6^, Wilcoxon signed-rank test; n = 26 mice). The dLight signal remained significantly elevated in Hit trials compared to Miss trials across the following 1-s period (Hit 1.49 ± 0.24, Miss −0.01 ± 0.04; p = 1.7 x 10^−5^, Wilcoxon signed-rank test; n = 26 mice).

In the Delayed reward task, the dLight dynamics (averaged across the 3 testing days) changed further and differed prominently across trial types (Fig. 2C). Whisker deflection in Hit trials evoked a large, apparently monophasic increase in dLight fluorescence decaying close to baseline before the end of the 1 s delay period, while licking largely remained at a constant high level during the delay period. Upon reward delivery in Hit trials, a second large increase in dLight fluorescence lasting around 1 s was robustly observed. In contrast, only a small brief phasic increase in fluorescence shortly after the whisker deflection was found in Miss trials on average followed by a small decrease relative to prestimulus baseline, apparently similar to the Miss trials observed in the Whisker detection task. Quantified across the 1 s period following whisker deflection relative to a baseline 0.5–0 s before the whisker stimulus, there was a significant difference in dLight signals comparing Hit and Miss trials (Hit 1.14 ± 0.19, Miss 0.02 ± 0.06; p = 2.1 x 10^−4^, Wilcoxon signed-rank test; n = 19 mice). In the following 1-s period, when reward was delivered in Hit trials, the dLight signals were also significantly different (Hit 2.30 ± 0.20, Miss 0.08 ± 0.05; p = 1.3 x 10^−4^, Wilcoxon signed-rank test; n = 19 mice).

In the Reward omission task, in which reward delivery was omitted in about half of the Hit trials, the dLight signals were again modified and differed across trial types, for example in the comparison of rewarded vs unrewarded Hit trials (Fig. 2D). On average, rewarded Hit trials here appeared similar to Hit trials in the Delayed reward task. Both whisker stimulus and reward delivery evoked large transient increases in dLight fluorescence in rewarded Hit trials. However, in unrewarded Hit trials only the whisker deflection evoked a large increase in dLight fluorescence, and in the absence of reward delivery during the reporting period, a strong decrease in dLight fluorescence relative to prestimulus baseline was observed. Quantified across the 1 s period following whisker deflection relative to a baseline 0.5–0 s before the whisker stimulus, we did not find any significant differences in dLight signals, suggesting the robustness of our measurements (Rewarded Hit 1.49 ± 0.26, Unrewarded Hit 1.64 ± 0.26; p = 0.08, Wilcoxon signed-rank test; n = 16 mice). During the next second, when reward was either delivered or not, the dLight signals diverged in Rewarded Hit trials compared to Unrewarded Hit trials (Rewarded Hit 2.62 ± 0.24, Unrewarded Hit −0.49 ± 0.30; p = 4.4 x 10^−4^, Wilcoxon signed-rank test; n = 16 mice).

In summary, analysis of different trial types revealed distinct dLight dynamics indicating clear task-related dopamine signals, which we further analyze below, aiming to untangle how putative sensory, motor and reward-related dopamine signals evolve across the learning of whisker-dependent goal-directed sensorimotor transformations.

### 2.3 Dopamine reward signals

**Fig. 3:**
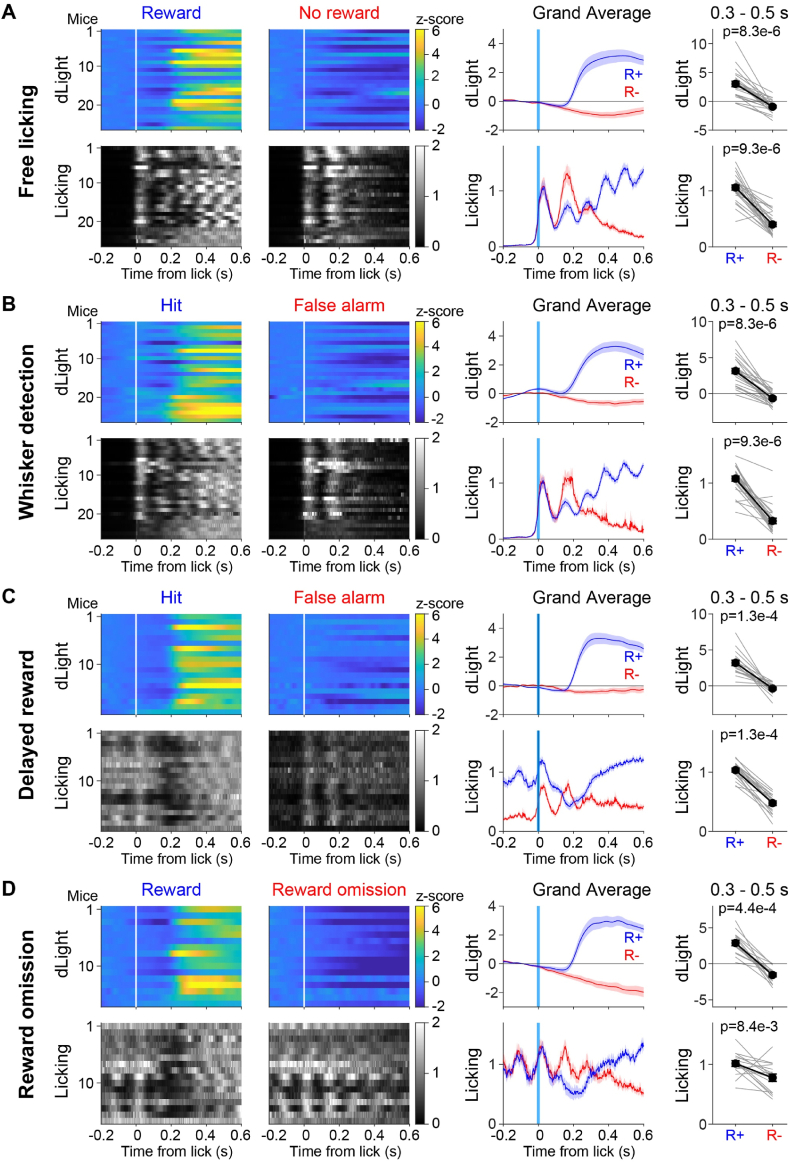
Dopamine reward responses to water acquisition. (**A**) Color-coded trial-averaged dLight fluorescence dynamics aligned to licking onset (white vertical bar at time 0 s) for each of the 26 mice recorded in the Free licking task for rewarded and unrewarded licking (upper left) (same data as shown in Fig. 2A, but now at higher temporal resolution). For the same mice, the licking power (normalized squared voltage measured by the piezo film) is indicated in grey for rewarded and unrewarded licking (lower left). The grand average dLight and licking dynamics across mice together with sem shading for rewarded (R+, blue) and unrewarded (R-, red) licking (center right). The average dLight fluorescence and licking in the 0.3–0.5 s period following lick onset was quantified for each mouse (grey lines) together with the mean ± sem (black lines and filled circles with error bars; Wilcoxon signed-rank test). (**B**) Similar to panel A, but for the Whisker detection task averaged across the three days of training and contrasting Hit trials with False alarm trials in which the mouse licked in the absence of whisker stimulus and therefore received no reward. Note here that the traces are aligned to the onset of licking and the pre-licking baseline has been subtracted, thus differing from Fig. 2B for the Hit trials. (**C**) Similar to panel B, but for the 19 mice in Delayed reward task, as before averaged across three days of training and aligned to licking onset in the reporting period 1–2 s after whisker deflection. (**D**) Similar to panel C, but for the 16 mice in the Reward omission task and aligned to licking onset in the reporting period 1–2 s after whisker deflection.

A large body of literature supports the hypothesis that rewards drive phasic increases in the firing of midbrain dopamine neurons leading to a transient increase in dopamine in the striatum most prominently in the firing of neurons in the VTA which project prominently to the nucleus accumbens [28,29]. Another important body of work suggests that dopamine signals are prominently related to movement [42]. We therefore investigated if we could distinguish reward-related from licking-related dLight signals. In order to compare trials with similar motor contributions, in this analysis we contrast Rewarded vs Unrewarded licking in the Free licking task (Fig. 3A), Hit vs False alarm trials in the Whisker detection task (Fig. 3B), Hit vs False alarm trials in the Delayed reward task (Fig. 3C), and Rewarded vs Unrewarded Hit trials in the Reward omission task (Fig. 3D).

Analyzing licking and dLight signals at high temporal resolution in rewarded and unrewarded trials of the Free licking task revealed interesting dynamics (Fig. 3A). The first tongue-spout contact signal from the piezo film was identical in rewarded and unrewarded licking trials on average. In rewarded trials, this first lick triggered valve opening, but the actual drop of water only appeared at the spout tip after the tongue had retracted in the rhythmical action of licking, typically at a frequency of around 10 Hz. It is thus when the tongue protruded for the second lick of the spout that mice encountered the water reward, in which case they applied less force to the spout with the tongue, presumably as they began to ingest the water. The dopamine signal began to rise almost immediately upon the mouse obtaining water, peaking at around 500 ms after the first tongue-spout contact. In unrewarded trials, we observed a different behavior. If the mouse did not encounter water reward on the second lick, it pushed more strongly on the lick spout presumably making sure that sufficient effort had been made to cross the threshold voltage on the piezo sensor needed to trigger reward delivery. In the absence of reward, the dLight signal decreased below the baseline. Quantified near the peak of the dLight response time from 300 ms to 500 ms after the first tongue-spout contact relative to a baseline period from 200 ms to 0 ms before the first tongue-spout contact there was a significant difference in the dLight signals between rewarded and unrewarded licking (Rewarded 3.03 ± 0.46, Unrewarded −0.93 ± 0.21; p = 8.3 x 10^−6^, Wilcoxon signed-rank test; n = 26 mice).

We similarly studied dLight and licking dynamics at high temporal resolution in the Whisker detection task averaged across the 3 days of training (Fig. 3B). Comparing rewarded Hit trials and unrewarded False alarm trials aligned to onset of licking, on average we found almost identical signals to those observed in response to rewarded and unrewarded licking in the Free licking task described above (Fig. 3A). The dLight signal increased rapidly after the second lick as the mouse received the drop of water on its tongue in Hit trials, but fell below baseline in the unrewarded False alarm trials. Licking dynamics were also similarly modulated as in the Free licking task. In the Whisker detection task, we also found a strong second lick in False alarm trials and prolonged licking in Hit trials. Quantified near the peak of the dLight response time from 300 ms to 500 ms after the first tongue-spout contact relative to a baseline period from 200 ms to 0 ms before the first tongue-spout contact, there was a significant difference in dLight signals between Hit and False alarm trials (Hit trials 3.15 ± 0.35, False alarm trials −0.66 ± 0.19; p = 8.3 x 10^−6^, Wilcoxon signed-rank test; n = 26 mice).

It was more difficult to directly compare the dLight and licking signals in the Delayed reward task, because already the strength of the first lick in the reporting period on average differed comparing Hit trials and False alarm trials, preventing unambiguous dissociation of motor signals (Fig. 3C). The results were nonetheless consistent with those presented above. The changes in dLight signal aligned to the first tongue-spout contact indicated a clear increase in response to water delivery in Hit trials, and a small decrease in dLight fluorescence in False alarm trials. Quantified near the peak of the dLight response time from 300 ms to 500 ms after the first tongue-spout contact in the delayed reporting period, relative to a baseline period from 200 ms to 0 ms before the first tongue-spout contact, there was a significant difference between dLight signals in Hit and False alarm trials (Hit trials 3.20 ± 0.36, False alarm trials −0.36 ± 0.18; p = 1.3 x 10^−4^, Wilcoxon signed-rank test; n = 19 mice).

Finally, the Reward omission task aimed at enabling direct comparison of rewarded and unrewarded trials with equal degrees of licking (Fig. 3D). Here, we focused our analyses on Hit trials in which the mouse received the brief single whisker stimulus at time 0 s and typically licked strongly during the 1 s delay period. The first lick after the onset of the delayed reward window, triggered reward delivery in rewarded Hit trials but no reward was delivered in unrewarded Hit trials. Aligned to the first lick in the reporting period, we again observed the stereotypical dLight and licking dynamics reported above. Licking strength decreased on the second lick in rewarded trials and was accompanied by a rapid increase in dLight fluorescence. In unrewarded trials, the mice continued licking rhythmically and indeed there were no cues to indicate reward omission trials beyond knowing the timing of the reward window from training in the Delayed reward task. A strong decrease in dLight fluorescence gradually developed during this unrewarded reporting period, perhaps reflecting accumulating evidence of a reward omission trial. Quantified near the peak of the dLight response time from 300 ms to 500 ms after the first tongue-spout contact in the delayed reporting period relative to a baseline period from 200 ms to 0 ms before the first tongue-spout contact there was a marked difference between dLight signals in Rewarded Hit and Unrewarded Hit trials (Rewarded Hit trials 2.89 ± 0.38, Unrewarded Hit −1.55 ± 0.27; p = 4.4 x 10^−4^, Wilcoxon signed-rank test; n = 16 mice).

In summary, the lick-triggered analysis supports the close correlation of increases in dLight fluorescence with obtaining reward, whereas increases in movement vigor without reward appeared to be correlated with decreased dLight fluorescence. Our data are therefore consistent with a prominent reward-related dopamine signal in the nucleus accumbens.

### 2.4 Water devaluation within behavioral sessions

**Fig. 4:**
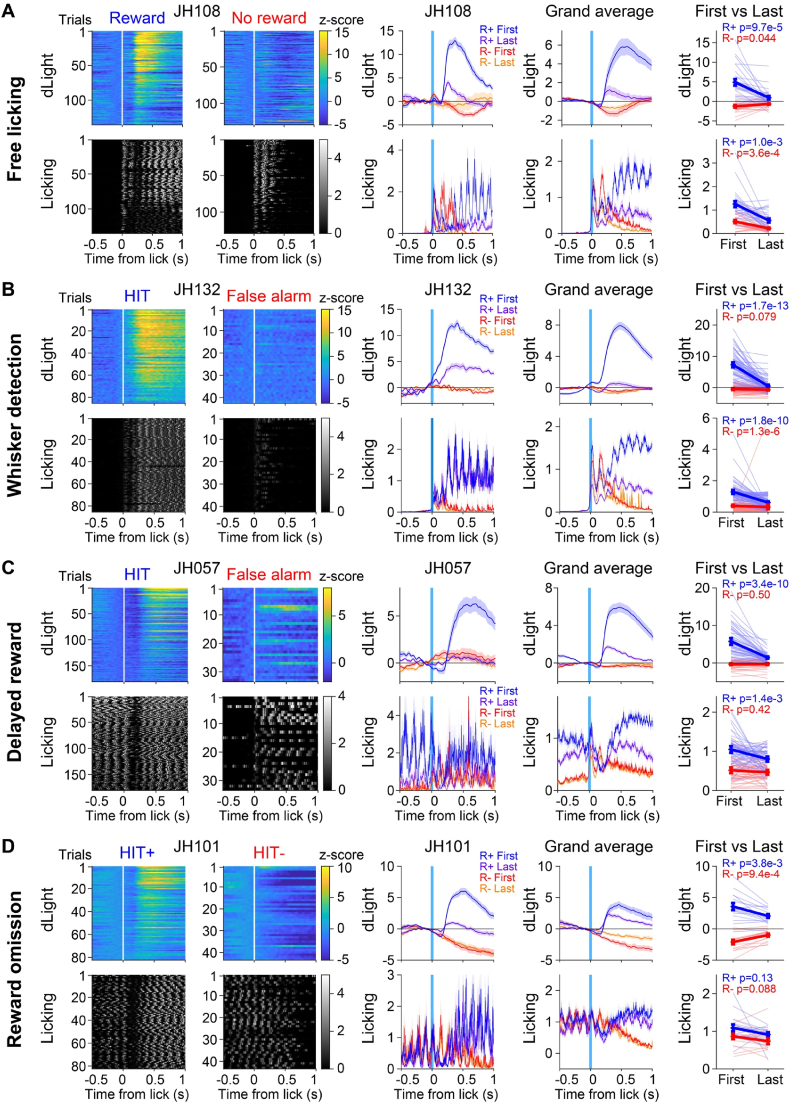
Devaluation of dopamine water reward signal within behavioral sessions. (**A**) An example session of the Free licking task from mouse JH108 showing each rewarded or unrewarded licking trial ordered separately according to time within the session with color-coded dLight dynamics and grey scale licking dynamics (left panels). The first and last 15 trials of rewarded and unrewarded licking were averaged (center panel) for rewarded trials (first 15 trials blue, last 15 trials magenta) and unrewarded trials (first 15 trials red, last 15 trials orange) of the example experiment, with grand average traces color-coded similarly across the 26 mice (center right). Quantification of the first and last 15 trials signal amplitudes at 0.2–0.4 s after licking onset for each mouse (far right; thin lines, blue for rewarded, red for unrewarded licking) together with the mean ± sem (thick lines and filled circles with error bars, blue for rewarded, red for unrewarded licking; Wilcoxon signed-rank test). (**B**) As panel A, except now for the Whisker detection task and showing example data from mouse JH132 on whisker detection task training day 3 contrasting Hit and False alarm trials (color-coded traces now indicate the first 15 Hit trials in blue, the last 15 Hit trials in magenta, the first 15 False alarm trials in red and the last 15 False alarm trials in orange). All data aligned to licking onset. (**C**) As panel B, but now for the Delayed reward task, showing example data from mouse JH057 on day 2 of the delayed reward task. (**D**) As panel C, but for the Reward omission task, showing example data from mouse JH101, and comparing rewarded Hit trials (HIT+) and unrewarded Hit trials (HIT-, i.e. reward omission trials).

Water is highly rewarding when thirsty, but not when sated. Mice obtained a large fraction of their daily water supply in their daily head-restrained training sessions. At the beginning of each behavioral session, the mice were thirsty and highly motivated to lick for water reward. Sessions ended when mice had largely stopped spontaneous and sensory-triggered licking, presumably because they were sated and no longer interested in obtaining more water. We therefore next investigated the within-session dopamine and licking dynamics comparing early trials with late trials in each session of mouse behavior. Aligned to the first lick time in the reporting window, in the Free licking task we investigated rewarded vs unrewarded licking trials (Fig. 4A), in the Whisker detection task and the Delayed reward task we examined Hit vs False alarm trials (Fig. 4B and C), and in the Reward omission task we quantified rewarded vs unrewarded Hit trials (Fig. 4D). In each task, there was an obvious decrease in the dLight reward signal as the mice accumulated water rewards within each session. Quantified around the peak of the reward window 300–500 ms after tongue spout contact, in the Free licking task the dLight response for rewarded licking decreased from 4.87 ± 0.82 over the first 15 rewarded trials compared to 0.93 ± 0.45 for the last 15 rewarded trials (p = 9.7 x 10^−5^, Wilcoxon signed-rank test; n = 26 mice). In the Whisker detection task, the dLight reward signal in Hit trials decreased from 7.30 ± 0.79 over the first 15 rewarded trials compared to 0.49 ± 0.45 for the last 15 rewarded trials (p = 1.7 x 10^−13^, Wilcoxon signed-rank test; n = 26 mice). In the Delayed reward task, the dLight reward signal in Hit trials decreased from 5.73 ± 0.88 over the first 15 rewarded trials compared to 1.44 ± 0.30 for the last 15 rewarded trials (p = 3.4 x 10^−10^, Wilcoxon signed-rank test; n = 19 mice). In the Reward omission task, the dLight reward signal in Rewarded Hit trials decreased from 3.56 ± 0.55 over the first 15 rewarded trials compared to 2.05 ± 0.30 for the last 15 rewarded trials (p = 0.0038, Wilcoxon signed-rank test; n = 16 mice).

Interestingly, the negative dopamine signals observed in non-rewarded lick trials also decreased in amplitude over the session. There was a significant difference in dLight signals between the first 15 and the last 15 non-rewarded lick trials in the Free licking task (First −1.25 ± 0.33; Last −0.67 ± 0.19; p = 0.044), as well as in the Reward omission task (First −2.1 ± 0.33; Last −0.98 ± 0.23; p = 9.4 x 10^−4^).

On average mice also decreased the vigor of licking across sessions, presumably also reflecting reduced motivation. In most sessions, the dLight reward signal appeared to decrease more rapidly than the decrease in licking vigor (as shown in the example experiment in Fig. 4A) and in some experiments the dLight signal decayed strongly without important change in licking vigor (as shown in the example experiments in Fig. 4B and D). The decrease in the dLight reward signal within sessions therefore appears consistent with a devaluation of water reward as thirst is quenched.

### 2.5 Sensory-evoked dopamine signals develop across days of learning

**Fig. 5:**
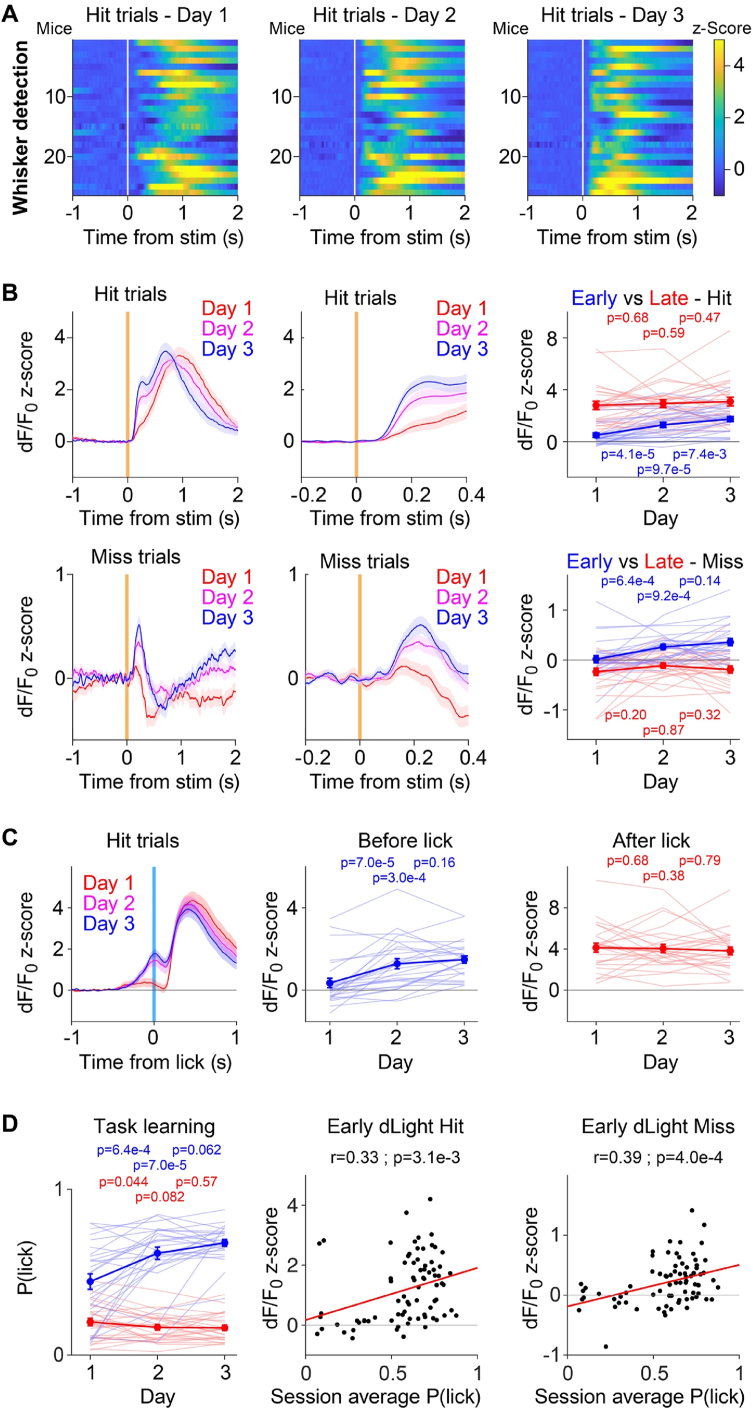
Development of a sensory-evoked dopamine signal across days of learning the whisker detection task. (**A**) Color-coded dLight fluorescence traces for each of the 26 mice carrying out the Whisker detection task averaged separately for each day of training aligned to the time of whisker deflection at time 0 s (white vertical line) and only including Hit trials. (**B**) Grand average dLight fluorescence traces across mice aligned to the time of the whisker deflection at time 0 s but separated according to the training day in the whisker detection task. Hit trials across days (upper left) are shown at higher temporal resolution (upper middle) with Miss trials below. The early response is quantified from 0.1 to 0.3 s after whisker deflection (right panels, blue) and a later period is quantified from 0.5 to 1.0 s after whisker deflection (right panels, red). Statistical comparisons across days were carried out with Wilcoxon signed-rank tests with Bonferroni corrected significance level of α = 0.017 (n = 26 mice). (**C**) Grand average dLight traces for Hit trials aligned to licking across the three days of training in the whisker detection task (left). The dLight signals were quantified for each mouse before the onset of licking from 0.1 to 0 s before the first tongue-spout contact (center, blue) and at the peak of the reward response from 0.3 to 0.5 s after the first tongue-spout contact (right, red). Statistical comparisons across days were carried out with Wilcoxon signed-rank tests with Bonferroni corrected significance level of α = 0.017 (n = 26 mice). (**D**) Most mice rapidly learned to lick in response to whisker deflection across the first three days of the Whisker detection task. Lick probability (left) was computed for trials with a whisker stimulus to compute the Hit rate (blue) and without a whisker stimulus to compute the False alarm rate (red) (light lines individual mice, dark lines with error bars mean ± sem). Statistical comparisons across days were carried out with Wilcoxon signed-rank tests with Bonferroni corrected significance level of α = 0.017 (n = 26 mice). For each mouse and each of the three days of training, we correlated the mean amplitude of the whisker deflection-evoked dLight signal in Hit trials (center), or Miss trials (right), with the session averaged Hit rate, finding significant positive Pearson correlations for both (n = 78 sessions).

In our experimental conditions, dLight signals in nucleus accumbens appear to largely report reward acquisition. A further prominent feature of dopamine signals supported by a large body of literature is that they emerge across learning in response to sensory cues that predict reward [28,29]. We therefore examined dopamine dynamics across the first three days of training in the Whisker detection task (Fig. 5). We investigated the two trial types in which the whisker deflection was delivered: i) Hit trials in which the mouse licked within 1 s of the whisker stimulus and received a reward and ii) Miss trials in which the same whisker deflection did not evoke licking and thus no reward was delivered. Aligned to the time of whisker deflection, across individual mice (Fig. 5A) and averaged across mice for each day of training in the Whisker detection task (Fig. 5B), we found an obvious increase across days in the first component of the dLight fluorescence signal evoked by whisker stimulus. In Hit trials, quantified from 100 ms to 300 ms after whisker deflection relative to a baseline from 200 ms to 0 ms before whisker stimulus, there was a significant increase in the early dLight response across training days (Hit trials early response: Day 1, 0.50 ± 0.17; Day 2, 1.29 ± 0.21; Day 3, 1.74 ± 0.19; Day 1 vs 2, p = 4.1 x 10^−5^; Day 1 vs 3, p = 9.7 x 10^−5^; Day 2 vs 3, p = 0.0074; Wilcoxon signed-rank test with Bonferroni corrected significance level of α = 0.017; n = 26 mice). At later times, as the mouse acquired the water droplet in Hit trials, there was a second increase in dLight fluorescence presumably reflecting the dopamine reward signal, and this appeared to be relatively stable in amplitude across training days. The fast time course of Hit trials with licking typically occurring around 300 ms after the whisker stimulus likely causes a mixing of sensory, motor and reward signals, which can be removed by exclusively analyzing Miss trials, in which the mouse fails to react with a motor response to the sensory stimulus. Interestingly, we also found a clear increase in a fast early dLight signal in Miss trials across days, although of a much smaller amplitude than the early response in Hit trials (Fig. 5B). Quantified from 100 ms to 300 ms after whisker deflection relative to a baseline from 200 ms to 0 ms before whisker stimulus, there was a significant increase in the early dLight response in Miss trials across training days (Miss trials early response: Day 1, 0.02 ± 0.08; Day 2, 0.26 ± 0.05; Day 3, 0.36 ± 0.07; Day 1 vs 2, p = 6.4 x 10^−4^; Day 1 vs 3, p = 9.2 x 10^−4^; Day 2 vs 3, p = 0.14; Wilcoxon signed-rank test with Bonferroni corrected significance level of α = 0.017; n = 26 mice). At later times, instead of the secondary increase in dLight signal found in Hit trials, we found a decrease in dLight relative to the prestimulus baseline in Miss trials. The analysis of both Hit and Miss trials aligned to the time of whisker deflection therefore indicates the development of a sensory response across days of task learning. Our data are therefore in good agreement with previous reports showing that dopamine transients develop across the associative learning of sensory cues which predict future reward.

In order to examine the late presumably reward-related dLight signal in Hit trials in further detail, we aligned trials to the onset of licking (Fig. 5C). Analyzed across days, there was an obvious increase in the pre-licking dLight signal, presumably resulting from the sensory-evoked response, whereas the later presumed reward-related dLight signal appeared on average to be stable across days. Quantified from 100 ms to 0 ms before tongue-spout contact relative to a baseline period from 1000 ms to 500 ms before tongue-spout contact, we found a significant increase in the prelicking sensory-related dLight signal (Hit trials prelicking signal: Day 1, 0.35 ± 0.23; Day 2, 1.28 ± 0.24; Day 3, 1.49 ± 0.18; Day 1 vs 2, p = 7.0 x 10^−5^; Day 1 vs 3, p = 3.0 x 10^−4^; Day 2 vs 3, p = 0.16; Wilcoxon signed-rank test with Bonferroni corrected significance level of α = 0.017; n = 26 mice). Quantified from 300 ms to 500 ms after tongue-spout contact relative to a baseline period from 1000 ms to 500 ms before tongue-spout contact, we found no significant change in the late reward-related dLight signal (Hit trials reward signal: Day 1, 4.13 ± 0.43; Day 2, 4.04 ± 0.41; Day 3, 3.81 ± 0.38; Day 1 vs 2, p = 0.68; Day 1 vs 3, p = 0.38; Day 2 vs 3, p = 0.79; Wilcoxon signed-rank test with Bonferroni corrected significance level of α = 0.017; n = 26 mice).

Whereas the sensory-evoked dLight signal increased across learning, the reward signal therefore appeared to be relatively constant across the three days of learning the Whisker detection task.

The changes in sensory-evoked dLight signals correlated with increased performance over days as mice learned that the whisker stimulus predicted reward availability (Fig. 5D). The Hit rate increased significantly across training days (Day 1, 0.44 ± 0.05; Day 2, 0.61 ± 0.04; Day 3, 0.68 ± 0.02; Day 1 vs 2, p = 6.4 x 10^−4^; Day 1 vs 3, p = 7.0 x 10^−5^; Day 2 vs 3, p = 0.06; Wilcoxon signed-rank test with Bonferroni corrected significance level of α = 0.017; n = 26 mice), without major changes in the False alarm rates (Day 1, 0.20 ± 0.02; Day 2, 0.17 ± 0.02; Day 3, 0.16 ± 0.02; Day 1 vs 2, p = 0.04; Day 1 vs 3, p = 0.08; Day 2 vs 3, p = 0.57; Wilcoxon signed-rank test with Bonferroni corrected significance level of α = 0.017; n = 26 mice). For each of the three training sessions, we correlated the session-averaged early dLight sensory response in Hit trials with the session-averaged Hit rate for each mouse finding a significant positive correlation (Slope 1.74; Pearson correlation coefficient = 0.33; p = 3.1 x 10^−3^) (Fig. 5D). Equally, we found a significant positive correlation for the session-averaged early dLight response in Miss trials versus the session-averaged Hit rate (Slope 0.69; Pearson correlation coefficient = 0.39; p = 4.0 x 10^−4^) (Fig. 5D). Thus, as mice learn to lick in response to the whisker stimulus across training sessions, there appears to be a concomitant increase in the fast sensory-evoked dLight signal.

### 2.6 Sensory-evoked dopamine signals within single learning sessions

**Fig. 6:**
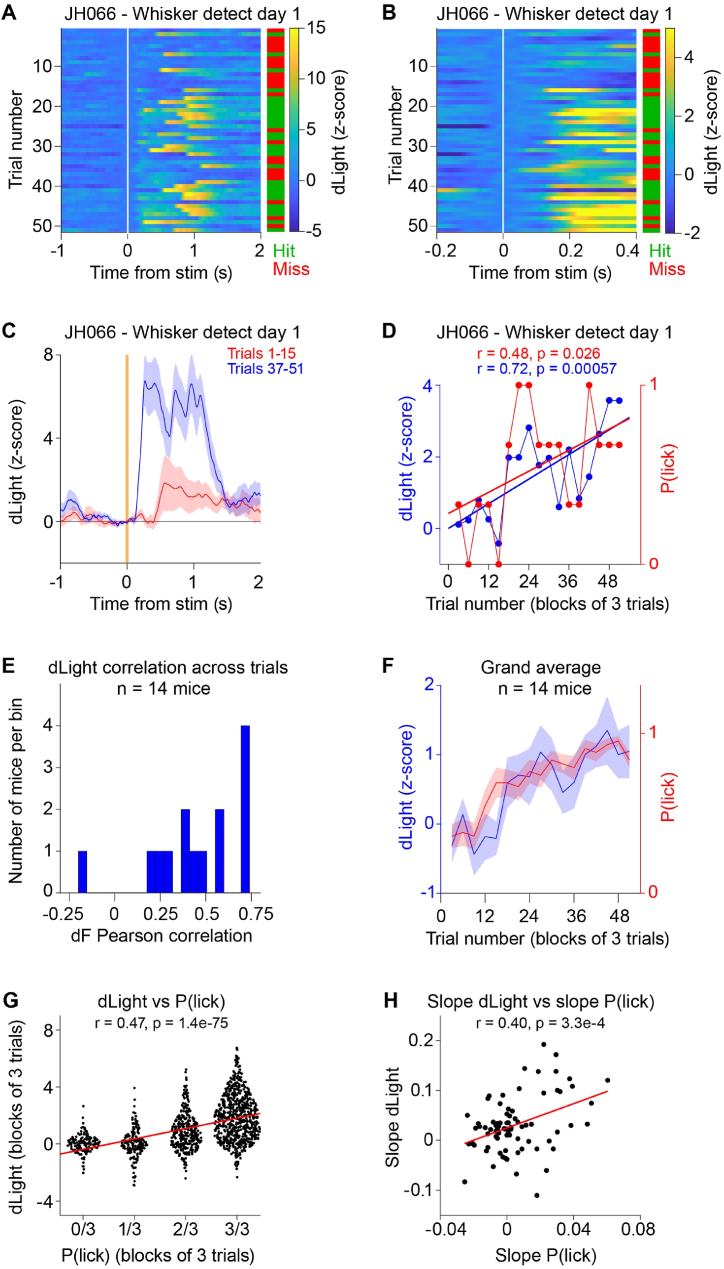
Single-session learning of the whisker detection task correlates with the development of sensory-driven dopamine signals. (**A**) Color-coded dLight dynamics for each of the first 51 whisker deflection trials for an example mouse JH066 on its first day of training in the Whisker detection task. Trials are aligned to the time of the whisker deflection at time 0 s. The green and red bar on the right indicates if the trial was a Hit (green) or Miss (red). (**B**) Same data as in panel A, but now shown at higher time resolution and on a different color scale to highlight the early sensory response. (**C**) The dLight dynamics across the averaged first 15 whisker trials (red) compared to the average of 15 later whisker trials (trials 37–51, blue) for the same example experiment as shown in panels A and B. (**D**) For the same example experiment as shown in panels A–C, averaged in blocks of three trials, the early dLight response quantified from 0.1 to 0.3 s after whisker deflection across the first 51 whisker trials (blue). Similarly averaged in blocks of three trials, the Hit rate across the same 51 whisker trials (red). Linear correlation revealed significant increase in dLight response across trials as the Hit rate also increased (Pearson correlation for dLight r = 0.72, p = 0.00057 and for Hit rate r = 0.48, p = 0.026). (**E**) Across the 26 mice that participated in the Whisker detection task, we selected the 14 mice for which there was a significant increase in Hit rate across the first 51 whisker trials (i.e. Pearson correlation p < 0.05) and we computed the dLight correlation across the same 51 trials. The distribution of Pearson dLight correlation coefficients is plotted as a histogram. (**F**) Grand average Hit rate (probability of licking, red) and dLight signal quantified 0.1–0.3 s after whisker deflection (blue) in blocks of 3 whisker trials across the first 51 whisker trials (same mice as panel E, i.e. the average of the 14 mice showing significant learning in the first 51 whisker trials). (**G**) For all mice and each block of three whisker trials across the first 51 whisker trials of each session of the three days of training in the Whisker detection task, we plot the amplitude of the early sensory-evoked response and correlate it with the Hit rate in each block of three whisker trials (Pearson correlation). (**H**) For the first 51 whisker trials of each session, we computed the slope of the sensory-evoked dLight signal as a function of whisker trial number and we also computed the slope of the Hit rate as a function of whisker trial number. The plot shows the slope of the dLight signal vs the slope of the Hit rate for each session (n = 78 sessions) and we found a significant positive Pearson correlation.

Having found clear evidence for learning-related increases in the early sensory-evoked dLight signal across days of the Whisker detection task, we next examined dLight changes within the sessions in which mice appeared to learn the task rule (Fig. 6). Visual inspection of the first ∼50 trials of individual Whisker detection sessions in which mice learned to lick the reward spout in response to the whisker deflection, suggested that an early dLight signal evoked by the whisker deflection might be acquired as the probability of licking increased. In data from an example mouse (Fig. 6A–D), on Day 1 of training in the Whisker detection task, mouse JH066 had a low Hit rate of 0.2 quantified across the first 15 whisker trials, whereas in whisker trials 37–51 the Hit rate had increased to 0.67 (Fig. 6A and B). The time course of the dLight signal averaged across whisker trials 1–15 compared to that of whisker trials 37–51 suggested the development of an early sensory response across single-session learning (Fig. 6C). The early dLight response was quantified across 100 ms–300 ms after whisker deflection in blocks of 3 whisker trials, and plotted together with the 3-trial block average of the probability of licking (Fig. 6D). The Hit rate increased in the first 51 whisker trials showing a significant correlation with trial number (Hit rate Pearson correlation r = 0.48, p = 0.026). Similarly, the early sensory dLight signal also increased across the first 51 whisker trials with a significant correlation with trial number (dLight Pearson correlation r = 0.72, p = 5.7 x 10^−4^) (Fig. 6D).

We next aimed to see if this pattern of changes could be observed across the population of mice studied. We found a significant correlation in Hit rate with trial number across the first 51 whisker trials in 14 out of the 26 mice participating in the Whisker detection task. Within this subgroup of mice in which we could identify a learning period, we quantified the correlation of the early dLight signal with trial number, finding a positive correlation in 13 out of the 14 mice (Fig. 6E). Averaging the Hit rate and dLight signals across the 14 mice showed an apparently near-simultaneous increase in the probability of licking in response to the whisker deflection and the amplitude of the early sensory-evoked dLight signal (Fig. 6F).

We further analyzed the early sensory-evoked dLight signal for 3-trial blocks as a function of the probability of licking in the same 3-trial blocks across the first 51 trials for each session and each mouse finding a significant correlation (Pearson correlation r = 0.47, p = 1.4 x 10^−75^, n = 1326 3-trial blocks across 78 sessions in 26 mice) (Fig. 6G). Finally, we correlated the slope of the early dLight response across the first 51 trials with the slope of the Hit rate across the first 51 trials for each session, again finding a significant correlation (Pearson correlation r = 0.40, p = 3.3 x 10^−4^, n = 78 sessions in 26 mice) (Fig. 6H). These analyses indicate that a fast sensory-evoked dLight signal emerged progressively with mice learning to lick in response to the whisker stimulus within single sessions.

## 3 Discussion

Here, through fiber photometry of the dopamine sensor dLight1.1 [38] in thirsty head-restrained mice, we report fast dopamine increases in nucleus accumbens driven by water reward delivery, as well as apparent dopamine decreases upon unrewarded licking. As mice learned to lick in response to whisker deflection, this reward-predicting sensory cue also drove a fast dopamine increase in nucleus accumbens. The precisely-defined behavioral paradigm enabled dopamine signals and licking to be measured with high temporal resolution longitudinally across different whisker-dependent detection task paradigms allowing us to begin to untangle sensory, motor, reward and motivation-dependent aspects across learning.

### 3.1 Water reward-related dopamine signals in nucleus accumbens of thirsty mice

The acquisition of water evoked a prominent fast phasic dopamine increase in the nucleus accumbens of thirsty mice (Fig. 3), but much less so in sated mice (Fig. 4). We found evidence consistent with the hypothesis that dopamine signals code for reward, with the strongest data comparing trials with or without reward delivery, but with similar licking movements, such as the rewarded vs unrewarded licking trials of the Free licking task (Fig. 3A) and the rewarded vs unrewarded Hit trials in the Reward omission task (Fig. 3D). However, it is important to note that we only monitored licking behavior and we cannot exclude contributions of the dLight signal relating to other reward-related changes in motor output such as reward-related facial expressions [35] or whole-body movements [43]. The fast dynamics of the dopamine water reward signal indicate a short-latency neuronal pathway from tongue somatosensation of water delivery to dopamine release in the nucleus accumbens. That the water reward signal is strongly reduced in sated mice suggests that this neuronal pathway is under important context-dependent control. Thirst is prominently represented by neuronal activity in the subfornical organ and the organum vasculosum of the lamina terminalis. These structures lack a blood-brain barrier, and neurons within these brain areas can directly sense blood osmolarity. A subset of neurons in the subfornical organ increase their activity in response to thirst and optogenetic stimulation of these neurons leads to rapid initiation of water-seeking behavior [44]. In turn neurons in the subfornical organ project to various downstream brain areas including the median preoptic nucleus in the hypothalamus. Neuronal activity in the median preoptic nucleus also correlates with thirst and optogenetic stimulation of these neurons also drives water-seeking behavior, apparently through inducing a negative valence state as a negative reinforcer against thirst [45,46]. Interestingly, neurons in the lateral hypothalamus might signal thirst and fluid intake to dopamine neurons in the VTA [47]. In future studies, it will be of great interest to further investigate the neuronal circuit mechanisms regulating how water reward acquisition drives a dopamine increase in nucleus accumbens of thirsty mice.

The dopamine reward signal might serve as a teaching signal for reinforcement learning of how to obtain water when thirsty. Upon being released the dopamine binds to G-protein coupled dopamine receptors expressed in different classes of neurons [48]. D1Rs expressed in MSNs in the nucleus accumbens have been proposed to play an important role in enhancing long-term potentiation of glutamatergic synaptic input onto MSNs [30,32]. Interestingly, the released dopamine can act to enhance plasticity of glutamatergic input that arrived within the last second [32]. The glutamatergic inputs which were active in the approximately 1-s preceding reward delivery therefore appear to be awarded an ‘eligibility trace’ through the retrospective enhancement of synaptic plasticity. Dopamine reward signals would therefore tend to enhance specifically the glutamatergic inputs which were active just before reward delivery onto D1R-MSNs in nucleus accumbens. The strengthening of these inputs could lead to similar patterns of accumbal neuronal activity in similar contexts, which in turn might lead to similar behavior, which might again result in reward acquisition causing further reinforcement. That inactivation of nucleus accumbens impaired task performance (Fig. S1) suggests that neuronal activity in nucleus accumbens participates in the execution of the whisker detection task, perhaps due to an overall general reduction in motivation and arousal [49], or perhaps more specifically due to perturbation of the representation of learned task rules including interactions with medial prefrontal cortex or hippocampus, which have also been shown to participate in the execution of the whisker detection task [17,21].

In good agreement with previous studies, we also found decreases in dopamine in response to unrewarded licking [[50], [51], [52], [53]]. In the Free licking task, we found a small reduction in dLight fluorescence preceding tongue-spout contact, which was further enhanced if no reward was delivered. Equally, in the Reward omission task, when mice were correctly licking in the delayed reporting period, there was a prominent reduction in dLight fluorescence in the reward omission trials. These data are consistent with effortful licking occurring at a cost reflected in decreased dopamine release in nucleus accumbens, which is reversed upon reward delivery causing increased dopamine in nucleus accumbens. Our data are also largely consistent with the reward prediction error hypothesis, in which the reward predicted to be delivered in Hit trials of the Delayed reward task, is disrupted in the Reward omission task, causing negative reward prediction errors, giving rise to negative dopamine signals. However, differing from the reward prediction error hypothesis, in our measurements, reward in thirsty mice was accompanied by dopamine increases, even when reward delivery was in principle fully predicted, although nonetheless dependent upon the voluntary initiation of licking. Other studies have found evidence for ramping dopamine signals towards the time of reward delivery [[54], [55], [56], [57]], but such activity was not prominent in our measurements in the Delayed reward task, where the dLight fluorescence decayed close to baseline during the delay period (Fig. 2C). Further experiments are therefore needed to more fully test various models with respect to the different whisker detection tasks studied here, and ultimately the causal neuronal circuits driving both positive and negative dopamine signals need to be determined.

### 3.2 Dopamine signals in nucleus accumbens across learning of the whisker detection task

Our data across Whisker detection task learning both across sessions (Fig. 5) and within sessions (Fig. 6) is consistent with previous studies reporting that sensory cues predicting future reward acquire fast-onset transient dopamine increases in nucleus accumbens across learning [28,29]. In future experiments, it will be important to investigate how the midbrain dopaminergic neurons in the VTA, which are presumably largely responsible for the dLight signals in nucleus accumbens, become responsive to whisker deflection across task learning. VTA neurons receive input from a large number of brain areas which could contribute to driving the learning-induced sensory excitation of midbrain dopamine neurons [58,59]. Monosynaptic rabies tracing suggests that both somatosensory and motor cortex innervate midbrain dopamine neurons, mainly in substantia nigra pars compacta, but also in VTA [59]. Cortical neurons could thus provide part of the excitatory signal driving a whisker deflection-evoked sensory signal in dopamine neurons upon learning of the Whisker detection task. However, there are many other possible pathways, including synaptic input from other parts of the basal ganglia, including the dorsolateral striatum, which responds to whisker stimulation [26,60,61] in a learning-dependent manner in the whisker detection task [25].

The dopamine released in the nucleus accumbens in response to reward-predicting whisker deflection upon learning may serve various functions. In the same way that the water reward dopamine signal can be used to reinforce rewarded sensory-to-motor transformations enhancing reward acquisition, the whisker deflection-evoked dopamine signal could serve the same purpose for second order conditioning. As a strongly-associated reward predictor, whisker deflection in itself could be considered rewarding, typically leading to better than expected immediate outcomes. Thus, a dopamine signal associated with whisker deflection could reinforce behaviors leading to enhanced probabilities of obtaining a whisker deflection through gating synaptic plasticity in nucleus accumbens. For example, in our tasks, mice had to learn to withhold licking in the seconds before trial initiation, otherwise the trials were aborted. The whisker deflection-evoked dopamine increase in nucleus accumbens could also serve a more direct role in task execution. In expert mice, the sensory-evoked dopamine increase arrives in nucleus accumbens before the initiation of licking. The dopamine rise could thus contribute to the initiation of licking, by the relatively fast actions which can be mediated by metabotropic receptors on the 100-ms timescale. Interestingly, in support of a motor-function for dopamine, optogenetic stimulation of dopamine neurons has been shown to evoke orofacial movements in naïve head-restrained mice via D1Rs in nucleus accumbens [35]. Dopamine acting on D1Rs has been suggested to increase the excitability of MSNs [62]. Enhanced firing of some D1R-MSNs could contribute to driving downstream circuits responsible for the initiation of licking. Whisker deflection-evoked dopamine signals in nucleus accumbens could thus help initiate licking on the relevant timescale of around 100 ms. It is important to note that dopamine acts upon a multitude of intracellular signalling pathways including actions regulating neurotransmitter release at presynaptic specialisations [63]. Future experiments should directly examine the possibility that fast actions of dopamine might directly contribute to the initiation of licking through measurement and perturbation of dopamine signalling in nucleus accumbens, and more generally across dorsal and ventral striatum.

### 3.3 Limitations and future perspectives

Our data were collected using one of the first genetically-encoded fluorescent dopamine sensors dLight1.1 [38], and many other fluorescent dopamine sensors that exhibit improved properties in various respects have since been developed [39,[64], [65], [66]]. In the future, it will be of great interest to test these new genetically-encoded fluorescent dopamine sensors under our behavioral conditions to investigate whether additional signals might be observed. It will also be of great importance to combine the measurement of dopamine in nucleus accumbens with the measurement of other aspects of brain function. Multifiber measurements [67] could provide spatiotemporal dynamics of dopamine signals across different parts of the basal ganglia and cortex, for example it would be interesting to compare nucleus accumbens dopamine signals with those in dorsomedial and dorsolateral striatum [50]. Furthermore, dual-color fiber photometry allows simultaneous measurement of signals from reporters based on both green and red fluorescent proteins, and thus dopamine measurements can be combined with photometric measurements of genetically-encoded calcium indicators or reporters of various other neurotransmitters, such as glutamate, acetylcholine, serotonin, and noradrenaline [66,[68], [69], [70], [71], [72]]. It will also be of paramount interest to measure neuronal activity electrophysiologically, for example with Neuropixels recordings [73], to examine the correlations between dopamine fluorescence signals and the precise timing of action potentials across various areas and classes of neurons in the mouse brain.

Most importantly, future studies must probe causal mechanisms through refined perturbation experiments. Recent technical developments have contributed to advancing the efficacy of optogenetic actuators for inhibiting neuronal activity [[74], [75], [76]] and neurotransmitter release [77], and it would therefore be of great interest to manipulate the dopamine signals with high spatiotemporal resolution in the nucleus accumbens [78,79], as well as more broadly across the dorsoventral axis of the striatum, to probe for their causal role in enhancing synaptic plasticity to drive reward-based learning of the whisker detection task, perhaps aiming to focus on detailed investigation of single-session learning. There therefore remain many open questions for exciting future investigations into the synaptic circuit mechanisms underlying the reward-based rule-learning of a simple sensory-to-motor transformation. It will also be of enormous interest to determine the neuronal circuitry driving the water reward dopamine signal, tracking activity from water sensation on the tongue to dopamine release in the nucleus accumbens, as well as how this is regulated by thirst.

## 4 Materials and methods

All experimental procedures were carried out in compliance with protocols approved by the Swiss Federal Veterinary Office under licenses VD1628 and VD3442.

### 4.1 Mice

In this project, we utilized 26 mice for dLight recordings and 6 mice for muscimol inactivation, encompassing both sexes, aged 6–10 weeks before surgery. The mice were primarily C57BL/6J mice (n = 26), but a few mice (n = 6) were A2a-Cre, without making use of Cre-expression. The mice were housed in a temperature-controlled environment with a 12-h reversed light/dark cycle, with access to food and water ad libitum, until behavioral training began.

### 4.2 Surgery, virus injection and implantation

The mice were anesthetized with isoflurane and subsequently positioned on a surgical platform equipped with ear bars. After securing the mice, the skull was exposed, and the bregma and lambda landmarks were identified to adjust the Z-axis height, ensuring the skull plane was level. A metal head-holder was glued to the skull with superglue and reinforced with dental cement. The site above the nucleus accumbens (NAc) was marked (AP +1.4 mm, ML -1.0 mm), followed by a craniotomy to allow for viral injection and fiber implantation. To express dLight, 500 nl of AAV-Syn-dLight1.1, a genetically encoded dopamine sensor (Patriarchi et al., 2018), was injected. Two preparations of the same vector were used: one was a gift from Lin Tian's lab at UC Davis with a titre of 2.75 x 10^13^ vg/ml, diluted 2:1, and another was purchased from VVF Zurich with a titre of 2.6 x 10^12^ vg/ml, used undiluted. The virus was injected into the NAc at coordinates AP +1.4 mm, ML -1.0 mm, DV -4.6 mm at a rate of 100 nl/min via a micropipette connected to a manual microinjector (Narishige). The glass pipette remained in place for 10 min post-injection. Subsequently, a 400 μm outer diameter optic fiber cannula (MFC_400/430–0.48_4 mm_ZF2.5(G)_FLT, Doric Lenses) was implanted 100 μm above the injection site (AP +1.4 mm, ML -1.0 mm, DV -4.5 mm).

### 4.3 Behavioral training

After at least four weeks of viral expression and a brief habituation to the head-restrained condition on the behavioral set-up, the mice were water restricted to 1 ml of water per day which they obtained mostly during the training sessions. The mice were trained sequentially in: i) Free licking sessions (2 days), ii) a Whisker detection task (3+ days), iii) a Delayed reward task (3+ days), and iv) a Reward omission task with ∼50 % omission rate (1 day). Fiber photometry recordings were performed throughout these sessions. Behavioral control and behavioral data collection were carried out with custom-written computer routines using a National Instruments board interfaced through Matlab (Mathworks).

Free Licking Task (2 days): water-restricted mice were trained to lick the water spout (detected by a piezoelectric film sensor attached to the spout) to obtain a water reward (5 μl of sweetened water including 2 % sucrose or 5 μl of unsweetened water). Most mice were rewarded with sucrose sweetened water, and the small subset of mice rewarded with unsweetened water had similar dLight responses. Rewards were delivered in 50 % of the cases when mice licked within 1-s reporting periods separated by a 0.5–0.6 inter-trial interval plus a 3.5–4.5 s ‘No lick’ period. Licking during the ‘No lick’ period aborted the trial.

Whisker Detection Task (3+ days): After two days of Free licking, the whiskers on the right side were trimmed, leaving only the C2 whisker. The mice were then introduced to a Whisker detection task. The right C2 whisker was either placed in a glass pipette attached to a piezo bender that delivered a single brief (40 ms) deflection to the C2 whisker, or an iron particle was attached to the whisker, and stimulated with a brief (5 ms) magnetic pulse. During the training sessions, trials with or without stimulus were randomly presented, with a maximum of three consecutive trials of the same type. The inter-trial interval was 8–13 s, and a 4.5–5.5 s ‘No lick’ period preceded trial initiation (licking during the ‘No lick’ period aborted trial onset). Following the trial initiation, there was a 1-s reward window. If the mouse licked during this window in a whisker stimulus trial, it was considered a ‘Hit’ trial and rewarded (5 μl of water or 2 % sucrose in water); no licking resulted in a 'Miss' trial. Conversely, licking in a trial without a whisker stimulus was classified as a ‘False alarm’ trial, and no licking as a ‘Correct rejection’ trial. Training continued for at least three days, until mice reached good performance (d’ > 1.0), with d' = Z(Hit rate) − Z(FA rate) where the function Z(p), p ∈ [0,1], is the inverse of the cumulative distribution function of the Gaussian distribution, with loglinear correction for extreme values of d’ [80]. Then mice progressed to the Delayed reward task.

Delayed Reward Task (3+ days): After training in the Whisker detection task, mice were introduced to a task with a delayed response window. The task structure mirrored the whisker detection task except for the introduction of a 1-s delay between the whisker stimulus (or trial onset in trials without whisker stimulus) and the reward window. Licking during this delay period was neither rewarded nor punished, and mice often licked throughout the delay period. But importantly, the reward was delivered only if the mice licked within the reward period in whisker stimulus trials.

Reward Omission Task (1 day): Following three days of Delayed reward task, mice were introduced to a scenario with reward omissions during stimulus trials. The task structure was identical to the Delayed reward task. At the beginning of this session, the mice underwent 50 trials (25 with and 25 without whisker stimulus) where each whisker stimulus trial was rewarded if the mouse licked in the reward window. Subsequently, the reward probability for stimulus trials was reduced to 50 %; hence, half of these trials did not result in a reward even if the mouse licked correctly. A lick in such a reward omission trial was termed a ‘Hit-' trial, while a correct response in rewarded stimulus trials was called a ‘Hit+' trial.

### 4.4 Fiber photometry

During the recordings, excitation light from LEDs at wavelengths of 465 nm and 405 nm (both from Doric Lenses) was transmitted through excitation filters in a fluorescence cube (FMCA_AE (405_E1 (460–490)_F1(500–550)_S)) and focused onto a patch cord (P82366-11 FCM-MF 1.25, Doric Lenses). This fiber patch cord was connected to the chronically implanted fiber, allowing emission light (500–550 nm) to be collected through the same fiber and directed onto a photoreceiver (Newport 2151, Doric Lenses). The excitation light was sinusoidally modulated at frequencies of 208.616 Hz and 500.679 Hz for the 465 nm and 405 nm lights, respectively. The collected raw signal was then demodulated by a fiber photometry console (Doric Lenses) to isolate contributions from the two excitation sources.

Simultaneously with fluorescence measurements, behavior-related data such as valve-opening signals, stimulation signals, and licking traces were recorded. All data were integrated into the same fiber photometry recording file. The sampling rate varied depending on the recording setups (12,224 Hz in one and 20,000 Hz in another). In pre-processing, all recordings were downsampled to a frequency of 1 kHz to standardize the data. Next, the least squares fitting of the 405 nm channel to the 465 nm channel was carried out to correct for any potential photobleaching and movement-related artifacts, which were minimal in the head-restrained recording conditions of the current experiments. The fitted 405 nm signal was subtracted from the 465 nm signal and divided by the fitted 405 nm signal to calculate the fractional change in fluorescence: dF/F_0_ = (465 nm - fitted 405 nm)/fitted 405 nm. This signal was then aligned with the behavioral data generated by data acquisition analog-to-digital converters (National Instruments) in Matlab.

### 4.5 Muscimol inactivation

The nucleus accumbens was pharmacologically-inactivated by injecting muscimol, a GABA_A_ receptor agonist, into mice trained in the Whisker detection task with a performance level of d’ > 1.0. The pharmacological inactivation experiment was performed on the same mouse over three consecutive days. The infusion was administered 30 min before the start of the Whisker detection task. On the first day of infusion, 100 nl of 0.5 mM muscimol was injected bilaterally into the NAc at a rate of 100 nl/min, totaling 200 nl. On the second day, 100 nl of Ringer's solution was similarly injected bilaterally into the NAc. On the third day, a mixture of 0.5 mM muscimol and Chicago Sky Blue was delivered in the same volumes and rates bilaterally into the NAc. Following the behavioral session on the third day of infusion, the mice were immediately perfused, and their brains were extracted for further anatomical analysis.

### 4.6 Anatomy

After the final behavior training session, the mice were perfused with paraformaldehyde (4 % in PBS), and their brains were sectioned into 100 μm-thick serial slices using a vibratome (Leica VT 100). The brain sections were then mounted using Vectashield mounting medium and imaged with a slide scanner (Olympus VS120). The coronal sections were visually aligned to a mouse brain atlas [81] to assess the level and location of the viral expression, the placement of the fiber, and the locations of the pharmacological injections labelled with Chicago Sky Blue.

### 4.7 Statistical analysis

Statistical analyses were performed using a combination of Wilcoxon signed-rank tests and Pearson correlation coefficients (Matlab implementations). The Wilcoxon signed-rank test was employed to assess the significance of differences between trial types. Pearson correlation coefficients were calculated to assess the relationships between trial number and Hit rates, as well as trial number and the amplitude of the dLight signals, providing insights into the learning dynamics and sensory-evoked responses.

## Data and code availability

The complete data set and Matlab analysis code are freely available at the Open Access CERN database Zenodo: https://doi.org/10.5281/zenodo.13306827.

## CRediT authorship contribution statement

**Jun Huang:** Writing – review & editing, Writing – original draft, Software, Methodology, Investigation, Formal analysis, Data curation, Conceptualization. **Sylvain Crochet:** Writing – review & editing, Supervision. **Carmen Sandi:** Writing – review & editing, Supervision, Funding acquisition, Conceptualization. **Carl C.H. Petersen:** Writing – review & editing, Writing – original draft, Visualization, Supervision, Resources, Funding acquisition, Formal analysis, Data curation, Conceptualization.

## Declaration of competing interest

The authors declare the following financial interests/personal relationships which may be considered as potential competing interests: Carl Petersen reports financial support was provided by 10.13039/100000001Swiss National Science Foundation. Carmen Sandi reports financial support was provided by 10.13039/100000001Swiss National Science Foundation. If there are other authors, they declare that they have no known competing financial interests or personal relationships that could have appeared to influence the work reported in this paper.
