# Supplementary material for: Dopamine dynamics in nucleus accumbens across reward-based learning of goal-directed whisker-to-lick sensorimotor transformations in mice
Source: Heliyon. 2024 Sep 11;10(18):e37831. doi: 10.1016/j.heliyon.2024.e37831 (PMC11422591; doi:10.1016/j.heliyon.2024.e37831)
Supplement: Multimedia component 1 [file mmc1.docx]

*Heliyon*

*Supplemental Information*

**Dopamine dynamics in nucleus accumbens across**

**reward-based learning of goal-directed whisker-to-lick sensorimotor transformations in mice**

Jun Huang, Sylvain Crochet, Carmen Sandi and Carl C.H. Petersen

Supplemental information consists of

Figure S1 – related to Figure 1

**Supplemental Figure S1**


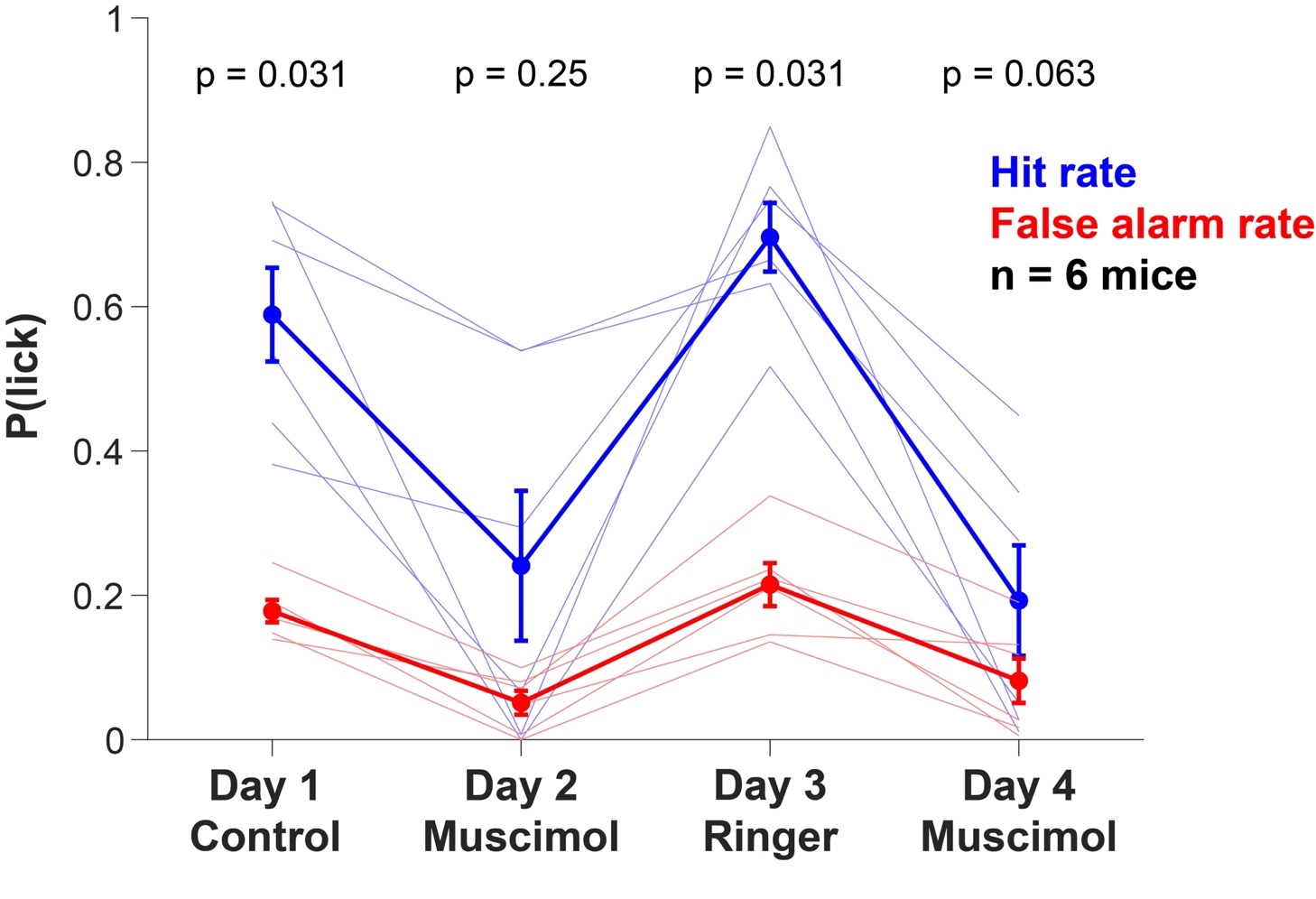


**Figure S1. Muscimol inactivation of nucleus accumbens impairs whisker detection performance, related to Figure 1.**

An additional 6 mice (not expressing dLight) were trained to lick the reward spout in response to deflection of the C2 whisker. Expert mice performed the whisker detection task on Day 1, which formed the baseline control day (Hit rate 58.9 ± 6.5%, False alarm rate 17.8 ± 1.6%, mean ± sem; p = 0.031, Wilcoxon signed-rank test; n = 6 mice). The next day, muscimol was injected bilaterally into the nucleus accumbens, which caused impaired detection task performance on Day 2 (Hit rate 24.1 ± 10.4%, False alarm rate 5.1 ± 1.7%; p = 0.25, Wilcoxon signed-rank test; n = 6 mice). A bilateral injection of Ringer’s solution into the nucleus accumbens on Day 3 acted as an injection control (Hit rate 69.6 ± 4.8%, False alarm rate 21.5 ± 3.0%; p = 0.031, Wilcoxon signed-rank test; n = 6 mice). Finally, muscimol was again injected bilaterally into the nucleus accumbens on Day 4 together with Chicago Sky Blue, again causing impairment in detection task performance (Hit rate 19.3 ± 7.7%, False alarm rate 8.2 ± 3.1%; p = 0.063 Wilcoxon signed-rank test; n = 6 mice). The mice were subsequently sacrificed, the brains extracted and sliced into coronal sections to verify injection into the nucleus accumbens. Hit rates and False alarm rates were significantly different on control Days 1 and 3, but not on the muscimol injection Days 2 or 4.

The Hit rate and False alarm rates were significantly lower during the sessions with muscimol injection compared to the sessions without muscimol (Control and Ringer): Hit rate with muscimol 21.7 ± 6.2% vs Hit rate without muscimol 64.3 ± 4.2%, mean ± sem, n = 12 sessions, p = 1.0x10^-4^, Wilcoxon rank sum test; False alarm rate with muscimol 6.6 ± 1.7% vs False alarm rate without muscimol 19.6 ± 1.7%, mean ± sem, n = 12 sessions, p = 2.2x10^-5^, Wilcoxon rank sum test.
